# Annotations

**Published:** 1911-04-29

**Authors:** 


### ANNOTATIONS.

### The General Medical Council Election.

So far there are two candidates in the field for the vacancy among the direct representatives on the General Medical Council caused by the death of Dr. L. S. McManus. Mr. George Jackson, whose election address was published last week, qualified in 1866 and is a Fellow of the Royal College of Surgeons of England, a vice-president (late president) of the Incorporated Medical Practitioners' Association, and a well-known Plymouth surgeon. He has served as a direct representative on the General Medical Council from 1902 to 1907, during which time he did good committee work and showed that the interests of the general practitioner were thoroughly safe in his hands. Dr. J. MacDonald, of Taunton, who is chairman of the Council of the British Medical Association and who qualified in 1887, is the other candidate. Mr. Jackson's claims seem to us to be superior to those of Dr. MacDonald, and he has the advantage of being an old member of the Council who is well acquainted with its procedure. There are many important questions before the medical profession at the present time, questions on which the Council, as the representative of the profession, will have to express an opinion. It is highly desirable, therefore, that general practitioners should carefully consider the matter of selecting a direct representative.

### Guardians and their Medical Officers.

It is refreshing to find that some Boards of Guardians in Ireland are willing to allow that under certain circumstances the labours of their medical officers are worthy of recognition. The Rathdown Guardians have granted an honorarium of £50 to Dr. Hamilton, medical officer of Arklow, in recognition of his devoted labours during a severe outbreak of typhus fever in his district. According to the *Journal of the Irish Medical Association*, which gives a full account of the meeting at which the honorarium was granted unanimously, Dr. Hamilton had to face unaided an outbreak of a particularly virulent type of typhus. Not only could he find no professional help in his combat with the disease, but lay aid was also wanting. This meant that the doctor had to visit the patient, help him into the ambulance, in some cases drive him to the hospital, and then help him up the stairs and put him to bed. Nor did the doctor's troubles end here, for when death came he had to lay out the body and coffin it. The hospital in which he laboured is an old, dilapidated building without drainage, water-closets, baths or water-supply; hospital workers need not be told what this means to a man struggling with a deadly disease such as typhus. The doctor's labours were crowned with success, despite the drawbacks under which he worked, and in some fourteen weeks the outbreak was quelled. It was then that Dr. Hamilton discovered what his losses really were. His patients had left him, some, perhaps, never to return. His pecuniary loss was great, and he applied to the Guardians for some extra allowance to make up for this, as well as to recompense him for the enormous strain put upon him by the outbreak. As stated above, he was granted £50, but this medical hero will have derived more pleasure from the congratulatory and appreciative words whereby it was accompanied than from the Guardians' cheque. We congratulate the Board on having such a medical officer.

### Medical Men and Social Reorganisation.

In an interesting article on the need for a general system of social medicine, published in the current number of that most excellent of American professional journals, the *Post-Graduate*, Professor Imbert, of Montpellier, pleads strongly for the systematic study of skilled labour by medical men. His thoughtful paper does not admit of quotation: in justice to the writer it should be read in its entirety. The study of labour in its various aspects is increasingly necessary if the medical profession is to retain its authoritative position with regard to questions involved in the discussion of compensation claims. At the present time, when so much money is squandered in the investigation of subjects of abstruse and theoretical interest, we hope that some lover of science will endow a scholarship for the study of the medical aspects of skilled labour. There is need for an investigation into the subject, and the results of such study are likely to be of great permanent and practical value.

### The London Degree.

In the course of his annual address as president of the Royal College of Physicians, Sir Thomas Barlow referred to the London degree for medical students, and strongly deprecated any attempt to lower the standard of the University's entrance examination. The manifest absurdities and inequalities of the London matriculation have been so often exposed that it would be a work of supererogation to lay stress on them here. At the same time it is necessary to protest against the allegation that any amendment in the syllabus of that examination necessarily signifies a lowering of the standard of entry. As the matriculation examination of the London University stands to-day it is a test which would have excluded Laennec, who could not remember a date in history, and Harvey, who "could not fancy ciphering." As it stands, again, it is a test which admits annually men who are incapable of writing a decently spelled and grammatically phrased letter in English, who are unacquainted with the rudiments of logical reasoning (notwithstanding the fact that the first six books of Euclid are compulsory), and whose ignorance of the classics or of foreign languages is a disgrace to the learned profession to which they belong. The test is too slight in some departments of knowledge, while it is far too high in one or two subjects; in other words, it is unequal. What the London student wants is a reasonably severe, intelligently arranged test, and the matriculation examinations of the provincial universities have a greater claim to be considered as such than the entrance examination which those who aspire to a degree in medicine at the London University must pass before they can commence their professional studies.

